# Endoscopic or Surgical Gastroenterostom for Malignant Gastric Outlet Obstruction: A Systematic Review and Meta-analysis

**DOI:** 10.1055/a-2904-9873

**Published:** 2026-07-13

**Authors:** Leonardo Marinho, Alessandrino T. de Oliveira, Márcio A. Barreira, Erica U. Holanda, Ana C. de Oliveira Monteiro

**Affiliations:** 1 Postgraduate Studies in Medical Sciences 28128 Universidade de FortalezaFortalezaCEBrazil; 2 Department of Surgery 633701 Dr José Frota InstituteFortalezaCEBrazil; 3 Department of Digestive Endoscopy 365090 General Hospital of FortalezaFortalezaCearáBrazil; 4 Department of Surgery 293278 Universidade Federal do Ceara Hospital Universitario Walter CantidioFortalezaCearáBrazil; 5 Department of Internal Medicine 672398 Hospital Geral Dr Waldemar AlcântaraFortalezaCEBrazil

**Keywords:** endoscopic ultrasonography, intervention EUS, endoscopy upper GI tract

## Abstract

**Background and study aims** Malignant gastric outlet obstruction (MGOO) is a debilitating condition requiring effective palliation. Surgical gastrojejunostomy (SGJ) has been the traditional standard but is associated with relevant morbidity. Endoscopic ultrasound-guided gastroenterostomy (EUS-GE) has emerged as a minimally invasive alternative, though comparative efficacy and safety remain incompletely defined. This systematic review and meta-analysis compared EUS-GE and SGJ for palliative management of MGOO.

**Methods** This review was registered in PROSPERO (CRD420261283768). MEDLINE, Embase, Cochrane CENTRAL, Scopus, and LILACS were searched from January 2015 to October 2025. Random-effects meta-analyses were performed using risk ratios for technical and clinical success, odds ratios for adverse events, serious adverse events, symptom recurrence, reintervention, and 30-day mortality, and mean differences for length of hospital stay. Prespecified subgroup and sensitivity analyses were conducted to assess the robustness of findings.

**Results** Ten studies (two randomized trials and eight observational studies) including 1,087 patients (659 EUS-GE, 428 SGJ) were analyzed. Technical success was comparable between groups. EUS-GE was associated with higher clinical success (RR 1.06; 95% CI 1.01–1.12), fewer overall AE (OR 0.35; 95% CI 0.22–0.56), and SAE (OR 0.52; 95% CI 0.28–0.93). EUS-GE resulted in lower symptom recurrence (OR 0.39; 95% CI 0.18–0.85) and shorter LOS (mean difference −4.11 days; 95% CI −5.29 to −2.92). Reintervention rates and 30-day mortality were similar.

**Conclusions** EUS-GE is a safe and effective minimally invasive alternative to SGJ for palliation of MGOO. These findings support the growing role of EUS-GE as a preferred palliative strategy in specialized centers.

## Introduction

Malignant gastric outlet obstruction (MGOO) is a clinical condition caused by a mechanical malignant blockage of the upper digestive tract at the level of the distal stomach, pylorus, or duodenum. 1 Malignant obstructions are predominant, with pancreatic cancer and metastatic invasion of the duodenum being the most common etiologies. 2

The patient usually presents as weak and malnourished due to not accepting oral nutrition. 1 The most common symptoms include vomiting, early postprandial fullness, persistent nausea, epigastric pain, dehydration, and weight loss. 3 This debilitating condition requires prompt intervention to alleviate symptoms, improve nutritional status, and enhance quality of life. 4

Considering the unresectable nature of such tumors, palliative therapy with a bypass procedure is often the first and, in many cases, only available therapeutic option. Surgical gastrojejunostomy (SGJ) is a classic and more invasive procedure, while endoscopic ultrasound-guided gastroenterostomy (EUS-GE) is a newer, technically complex, and less invasive procedure. Endoscopic enteral stenting is another therapeutic option that should be considered, especially for patients with poor prognosis and limited life expectancy. 1 4

Although SGJ has traditionally been considered the standard palliative approach for MGOO, it is associated with substantial morbidity, require an optimum nutritional status, prolonged hospitalization, and potential delays in systemic oncologic therapy. In recent years, EUS-GE has emerged as a minimally invasive alternative, enabled by advances in interventional EUS and the development of lumen-apposing metal stents (LAMS). Despite its increasing adoption in specialized centers, the role of EUS-GE in routine clinical practice remains controversial. 5

EUS-GE is a less invasive procedure that provides a robust bypass, and the endoscopic nature of the EUS-GE procedure may offer significant safety advantages over a traditional surgical bypass. However, it is not commonly used. This is likely due to its technically challenging nature compared to other interventional endoscopic procedures, coupled with a lack of standardization of the technique and the need for specialized centers. 6

Current evidence is largely derived from retrospective cohorts, with limited randomized data and heterogeneous outcome reporting. As a result, there is no consensus regarding the optimal palliative approach for MGOO, and clinical decision-making often relies on local expertise rather than high-certainty evidence. It is therefore necessary to better guide clinical practice and guideline development.

In palliative care patients, the choice of surgical technique focuses on improving quality of life and reducing suffering. This systematic review and meta-analysis aimed to compare the efficacy and safety of EUS-GE versus SGJ for palliative management of MGOO. Primary outcomes were technical success, clinical success, and adverse events (AE). Secondary outcomes included serious AEs (SAE), symptom recurrence, reintervention, length of hospital stay (LOS), and mortality. Prespecified subgroup, sensitivity, and exploratory meta-regression analyses were conducted to assess the robustness of findings and investigate potential sources of heterogeneity.

## Materials and Methods

### Protocol and Registration

The systematic review and meta-analysis protocol has been registered in the International Prospective Register of Systematic Reviews (PROSPERO) under CRD420261283768. This study was performed per the recommendations of the Cochrane Handbook of Systematic Reviews of Interventions and the Preferred Reporting Items for Systematic Reviews and Meta-analysis (PRISMA) guidelines. 7

### Search Strategy

A comprehensive literature search was conducted in the MEDLINE (PubMed), Embase, Cochrane CENTRAL, Scopus, and LILACS from January 2015 to October 2025. The search was supplemented by screening clinical trial registries (ClinicalTrials.gov and WHO-ICTRP) to identify unpublished or ongoing studies.

Search terms were based on a combination of Medical Subject Headings, Entree terms, and free-text keywords related to gastric outlet obstruction, gastrojejunostomy, and ultrasound-guided techniques. Boolean operators (“AND,” “OR”) were applied, and the search strategy was tailored to each database. The search strategy was limited to studies involving patients with malignant disease and only articles published in English were included.

### Eligibility Criteria

Comparative studies were considered eligible, including randomized controlled trials (RCTs) and observational studies conducted in single-center or multicenter settings, enrolling adult patients.

Studies were excluded if they lacked a direct comparator, were case reports or case series without a control group, narrative reviews, systematic reviews or meta-analyses, editorials, or if they included fewer than 10 patients per study arm. Studies involving pediatric populations, exclusively benign obstructions, or those that did not report separate data for malignant etiologies were also excluded. In cases of overlapping cohorts or duplicate publications, the most comprehensive and/or most recent study was retained.

Two reviewers independently screened titles, abstracts, and full texts for eligibility. Data extraction was independently performed using a standardized data collection form. Disagreements were resolved by consensus or, when necessary, by consultation with a third reviewer.

The study selection process is presented in the PRISMA flow diagram.

### Outcomes

The primary outcomes were clinical success, technical success, and AE.

Clinical success was defined as the ability to tolerate oral intake without vomiting and/or improvement in the Gastric Outlet Obstruction Scoring System (GOOSS). The GOOSS is a validated scale ranging from 0 to 3, defined as follows: 0 = no or inadequate oral intake; 1 = liquids or thickened liquids; 2 = semi-solids or low-residue diet; and 3 = full, unmodified diet. 8

Technical success was defined as the successful creation of a gastroenteric anastomosis using the initially intended technique, as reported by the original study.

AEs were systematically extracted and defined according to the original criteria and methodologies of each included study. When reported, severity was classified using established systems, including the Clavien–Dindo classification, which grades postoperative complications based on the level of intervention required, 9 and the ASGE Lexicon, which standardizes the reporting of endoscopy-related AEs according to severity and clinical consequences. 10

Secondary outcomes included SAE, 30-day mortality, symptom recurrence, reintervention rate, and LOS.

*SAEs* were defined as events classified as Grade III or higher according to the Clavien–Dindo classification 9 or as severe or fatal according to the ASGE lexicon. 10

30-day mortality was defined as death from any cause occurring within 30 days following the procedure.

Symptom recurrence was defined as the return of GOO-related symptoms after initial clinical success.

Reintervention rate was defined as the need for any additional endoscopic, radiological, or surgical procedure following the index intervention.

LOS was defined as the total duration of hospitalization, from admission to discharge, measured in days.

Follow-up duration was defined according to each included study and extracted as reported.

### Interventions

SGJ was defined as an anastomosis between the stomach and the small intestine performed via laparotomy, laparoscopy or robotic approach. EUS-GE was performed using LAMS to create a bypass of the gastrointestinal obstruction. For descriptive reporting in Table 1 , EUS-GE techniques were categorized as direct or device-assisted according to the classification proposed by Yang, Huang, and Cheng. 11

**Table 1:** Baseline characteristics of included studies.

First author, year	Sample size, n	Study design	Age, years, mean (SD)	Male, *n* (%)	Most common etiologies	Intervention procedure
Abbas, 2021	**EUS-GE** (25)	Single-center retrospective cohort	**EUS-GE** 65.3 (10.2)	**EUS-GE** 11 (44%)	Pancreatic cancer	**EUS-GE** Direct puncture technique with LAMS
	**SGJ** (27)		**SGJ** 61.5 (14.8)	**SGJ** 13 (48%)		**SGJ** Laparoscopic and open techniques
Bang, 2025	**EUS-GE** (38)	Multicenter RCT	**EUS-GE** 65.3 (15.1)	**EUS-GE** 23 (60.5%)	Pancreaticobiliary cancer	**EUS-GE** Direct puncture technique with LAMS
	**SGJ** (36)		**SGJ** 61.5 (12.3)	**SGJ** 21 (58.3%)		**SGJ** Laparoscopic, robotic, and open techniques
Canakis, 2023	**EUS-GE** (187)	Multicenter retrospective study	**EUS-GE** 67.50 (12.6)	**EUS-GE** 111 (59.4%)	Pancreatic cancer	**EUS-GE** Direct puncture technique with LAMS
	**SGJ** (123)		**SGJ** 61.60 (13.5)	**SGJ** 55 (44.7%)		**SGJ** Laparoscopic and open techniques
Chan, 2022	**EUS-GE** (30)	Retrospective two-center study	**EUS-GE** 63 (13.7)	**EUS-GE** 22 (73%)	Gastric and pancreatic cancer	**EUS-GE** EPASS
	**SGJ** (35)		**SGJ** 67.7 (9.9)	**SGJ** 23 (65%)		**SGJ** Laparoscopic technique
Jaruvongvanich, 2026	**EUS-GE** (232)	Two-center retrospective cohort	**EUS-GE** 64.5 (12.3)	**EUS-GE** 135 (58.2)	N/A	**EUS-GE** Direct puncture with LAMS, Guidewire-assisted, EPASS, Balloon catheter, and Pediatric gastroscope techniques
	**SGJ** (73)		**SGJ** 62.1 (14.4)	**SGJ** 36 (49.3)		**SGJ** Laparoscopic and open techniques
Khashab, 2016	**EUS-GE** (30)	Multicenter retrospective study	**EUS-GE** 70 (13.3)	**EUS-GE** 17 (57%)	Pancreatic cancer	**EUS-GE** Direct puncture with LAMS, Balloon-assisted EUS-GE, and EPASS
	**SGJ** (63)		**SGJ** 68 (9.6)	**SGJ** 32 (52%)		**SGJ** Open technique
Kouanda, 2021	**EUS-GE** (36)	Single-center retrospective cohort study	**EUS-GE** 70.4 (11.8)	**EUS-GE** 20 (55%)	Gastric and pancreatic cancer	**EUS-GE** Direct puncture technique with LAMS
	**SGJ** (14)		**SGJ** 71.5 (15.6)	**SGJ** 8 (57%)		**SGJ** Open technique
Martinet, 2024	**EUS-GE** (56)	Multicenter retrospective study	**EUS-GE** 70.1 (14.4)	**EUS-GE** 32 (57.1%)	Bilio-duodenal-pancreatic cancer	**EUS-GE** Direct puncture with LAMS and Wireless endoscopic simplified technique
	**SGJ** (41)		**SGJ** 67 (16.9)	**SGJ** 25 (61%)		**SGJ** Open technique
Pavert, 2025	**EUS-GE** (48)	Multicenter RCT	**EUS-GE** 69.4 (9.2)	**EUS-GE** 28 (58%)	Pancreatic cancer	**EUS-GE** Direct puncture technique with LAMS
	**SGJ** (50)		**SGJ** 70.6 (10.5)	**SGJ** 27 (54%)		**SGJ** Laparoscopic technique
Pawa, 2023	**EUS-GE** (29)	Retrospective study	**EUS-GE** 66.3 (12.6)	**EUS-GE** 19 (65%)	Pancreatic cancer	**EUS-GE** Direct puncture technique with LAMS
	**SGJ** (15)		**SGJ** 64.1 (13.7)	**SGJ** 8 (46%)		**SGJ** Robotic technique

SD: standard deviation; RCT: randomized controlled trial; n: number; SGJ: surgical gastrojejunostomy; EUS-GE: EUS-guided gastroenterostomy. N/A: not available; EPASS: EUS-guided balloon-occluded gastrojejunostomy bypass; LAMS: lumen-apposing metal stent.

### Risk of Bias Assessment

The risk of bias was independently assessed by two reviewers. RCTs were evaluated using the Cochrane Risk of Bias 2 (RoB 2) ( Table 2 ), 12 whereas observational studies were assessed using the ROBINS-I tool ( Table 3 ). 13

**Table 2:** Risk of bias assessment for randomized studies (RoB 2.0).

Study	Bias from randomization process	Bias due to deviations from intended interventions	Bias due to missing outcome data	Bias in measurement of the outcomes	Bias in selection of the reported result	Overall risk of bias
Bang et al. 2025	Low	Some concerns	Some concerns	Low	Low	Some concerns
ENDURO 2025	Low	Some concerns	Some concerns	Some concerns	Low	Some concerns

**Table 3:** Risk of bias assessment for nonrandomized studies (ROBINS-I).

Study	Bias due to confounding	Bias in selection of participants	Bias in classification of interventions	Bias due to deviations from intended interventions	Bias due to missing data	Bias in measurement of outcomes	Bias in selection of the reported result	Overall risk of bias judgment
Abbas 2022	Serious	Serious	Low	Moderate	Moderate	Moderate	Low	Serious
Canakis 2023	Serious	Serious	Moderate	Moderate	Moderate	Low	Low	Serious
Chan 2023	Serious	Moderate	Low	Moderate	Low	Moderate	Low	Serious
Jaruvongvanich 2023	Moderate	Serious	Low	Moderate	Moderate	Low	Low	Serious
Khashab 2017	Serious	Moderate	Low	Moderate	Moderate	Low	Low	Serious
Kouanda 2021	Serious	Moderate	Low	Low	Moderate	Moderate	Low	Serious
Martinet 2024	Serious	Moderate	Low	Low	Low	Moderate	Low	Serious
Pawa 2023	Serious	Serious	Low	Low	Moderate	Moderate	Low	Serious

### Certainty of evidence (GRADE)

The certainty of evidence for each outcome was assessed using the grading of recommendations assessment, development, and evaluation (GRADE) approach ( Table 4 ). 14

**Table 4:** GRADE Summary of Findings.

Outcome (effect measure)	Number of studies	Study design	Risk of bias	Inconsistency	Indirectness	Imprecision	Other considerations	Number of patients (EUS-GE)	Number of patients (SGJ)	Effect (95% CI)	Certainty of the evidence (GRADE)
Technical success (RR)	9	NRS and RCTs	Serious ^a^	Not serious	Serious ^d^	Not serious	Egger’s test showed no statistically significant asymmetry	621	392	RR 0.98 (0.96–0.99)	⨁⨁⨁◯ Moderate ^a,d^
Clinical success (RR)	10	NRS and RCTs	Serious ^a^	Serious ^b^	Serious ^d^	Not serious	Egger’s test showed no statistically significant asymmetry	659	428	RR 1.06 (1.01–1.12)	⨁⨁⨁◯ Moderate ^a,b,d^
Adverse events (OR)	10	NRS and RCTs	Serious ^a^	Serious ^b^	Serious ^d^	Not serious	Strongly suspected publication bias ^f^ ; Strong association ^g^	659	428	OR 0.35 (0.22–0.56)	⨁⨁◯◯ Low ^a,b,d,f,g^
Serious adverse events (OR)	8	NRS and RCTs	Serious ^a^	Serious ^b^	Serious ^d,i^	Not serious	Egger’s test showed no statistically significant asymmetry	427	371	OR 0.52 (0.28–0.93)	⨁⨁◯◯ Low ^a,b,d,i^
Rate of re-intervention (OR)	10	NRS and RCTs	Serious ^a^	Very serious ^c^	Serious ^d,i^	Serious ^e^	Funnel plot showed wide dispersion of studies ^h^ ; Egger’s test showed no statistical evidence of asymmetry	659	428	OR 0.58 (0.19–1.76)	⨁◯◯◯ Very low ^a,c,d,e,h,i^
Recurrence of symptoms (OR)	6	NRS and RCTs	Serious ^a^	Not serious	Serious ^d^	Not serious	Egger’s test showed no statistical evidence of asymmetry; Strong association ^g^	202	212	OR 0.39 (0.18–0.85)	⨁⨁⨁◯ Moderate ^a,d,g^
30-Day mortality (OR)	5	NRS and RCTs	Serious ^a^	Not serious	Serious ^d^	Serious ^e^	Egger’s test showed no statistical evidence of asymmetry	163	183	OR 0.73 (0.30–1.79)	⨁⨁◯◯ Low ^a,d,e^
Mean length of hospitalization (MD)	10	NRS and RCTs	Serious ^a^	Very serious ^c^	Serious ^d^	Serious ^e^	Strongly suspected publication bias ^f^	659	428	MD –4.11 (−5.29 to −2.92)	⨁◯◯◯ Very low ^a,c,d,e,f^

CI, confidence interval; MD, mean difference; RR, risk ratio; NRS, nonrandomized studies; RCT, randomized clinical trial; EUS-GE, endoscopic ultrasound-guided gastroenterostomy; SGJ, surgical gastrojejunostomy.

**Explanations:**

^a^ Many of the included studies did not statistically correct or propensity score match the important baseline difference amongst groups.

^b^ Moderate heterogeneity.

^c^ Significant heterogeneity.

^d^ Variations in intervention techniques.

^e^ Large confidence interval.

^f^ Egger’s plot and funnel plot visualization suggested publication bias present.

^g^ Due to the strong association (OR < 0.5) and concordance among studies, this evidence was upgraded in one point.

^h^ More likely related to methodological heterogeneity rather than publication bias (definition and report varied widely across studies).

^i^ The leave-one-out sensitivity analysis resulted in a change in statistical significance.

### Ethics Statement

Ethical approval was not required as this study was based on previously published data.

### Statistical Analysis

All statistical analyses were performed to compare the efficacy and safety of EUS-GE and SGJ. Given the predominance of observational studies and expected clinical/methodological heterogeneity, random-effects models were used throughout (main, subgroup, sensitivity, and meta-regression analyses), regardless of the observed statistical heterogeneity.

For dichotomous outcomes, pooled estimates were calculated using inverse-variance random-effects meta-analysis with the “metabin” function from the “meta” package in R. Risk ratios were used for technical and clinical success outcomes due to the high event rates, whereas odds ratios (ORs) were used for AEs, SAEs, symptom recurrence, reintervention, and 30-day mortality; this choice was kept consistent across main, subgroup, and sensitivity analyses.

For continuous outcomes, pooled mean differences (MDs) were estimated using the “metacont” function. Between-study variance (τ ^2^ ) was estimated using the restricted maximum-likelihood estimator, and confidence intervals for τ ^2^ were calculated using the Q-profile method. A continuity correction of 0.5 was applied in studies with single-zero event cells when applicable, while studies with zero events in both groups did not contribute to pooled relative effect estimates under the inverse-variance framework. Additional sensitivity analyses were performed using the Hartung–Knapp adjustment for random-effects models.

When studies reported median and interquartile range, these values were converted to mean and standard deviation according to the method of Wan et al., 15 enabling inclusion in the meta-analysis.

Statistical heterogeneity was assessed using the *I* ^2^ statistic, with values interpreted as follows: 0–25% (low heterogeneity), 26–50% (moderate heterogeneity), 51–75% (substantial heterogeneity), and >75% (considerable heterogeneity). In addition, heterogeneity was further assessed using Cochran’s Q test, with a *p* -value < 0.10 considered indicative of statistically significant heterogeneity.

Subgroup analyses were conducted according to study design (RCTs vs. observational studies) to explore potential sources of heterogeneity. Differences between subgroups were assessed using the test for subgroup differences, with *p* < 0.05 considered statistically significant.

All statistical tests were two-tailed, and a *p* -value < 0.05 was considered statistically significant for pooled effect estimates.

Sensitivity analyses assessed robustness by excluding studies meeting a stringent high-risk pattern (≥5 domains rated as moderate/serious). In addition, influence diagnostics using leave-one-out analyses and Cook’s distance were performed, and sensitivity analyses were repeated after exclusion of studies identified as influential. Univariate exploratory meta-regressions were conducted to investigate potential sources of heterogeneity when feasible, including available clinical and methodological study-level covariates. Publication bias was assessed by visual inspection of funnel plots and by Egger's test when applicable.

The quantitative synthesis of the outcomes was conducted in R software (version 4.4.3), mainly using the meta and metafor packages, adopting a significant level of 5% (α = 0.05) and 95% confidence intervals.

## Results

**Fig. 1:**
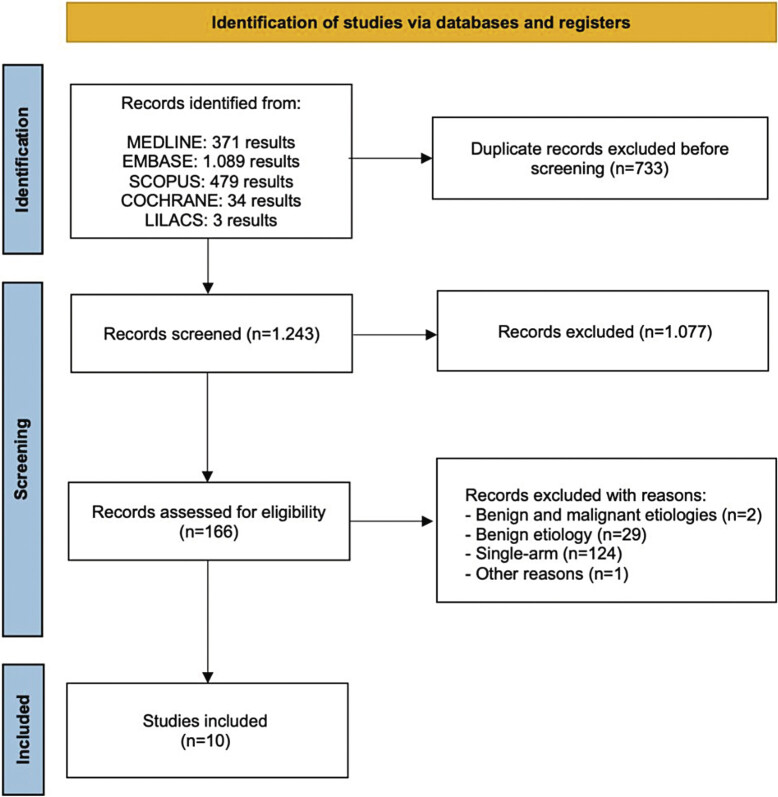
PRISMA flow diagram. 7

A total of 10 studies were included in the final analysis, comprising eight observational studies and two RCTs. Notably, the total number of patients included in RCTs ( *n* = 172) was substantially smaller than that of observational studies ( *n* = 915), which contributed the majority of the overall sample size. Overall, 1087 patients underwent endoscopic treatment for MGOO, including 659 treated with EUS-GE and 428 treated with SGJ. The study selection process is summarized in the PRISMA flow diagram ( Fig. 1 ).

The characteristics of the observational studies included in the meta-analysis are presented in Table 1 .

**Fig. 2:**
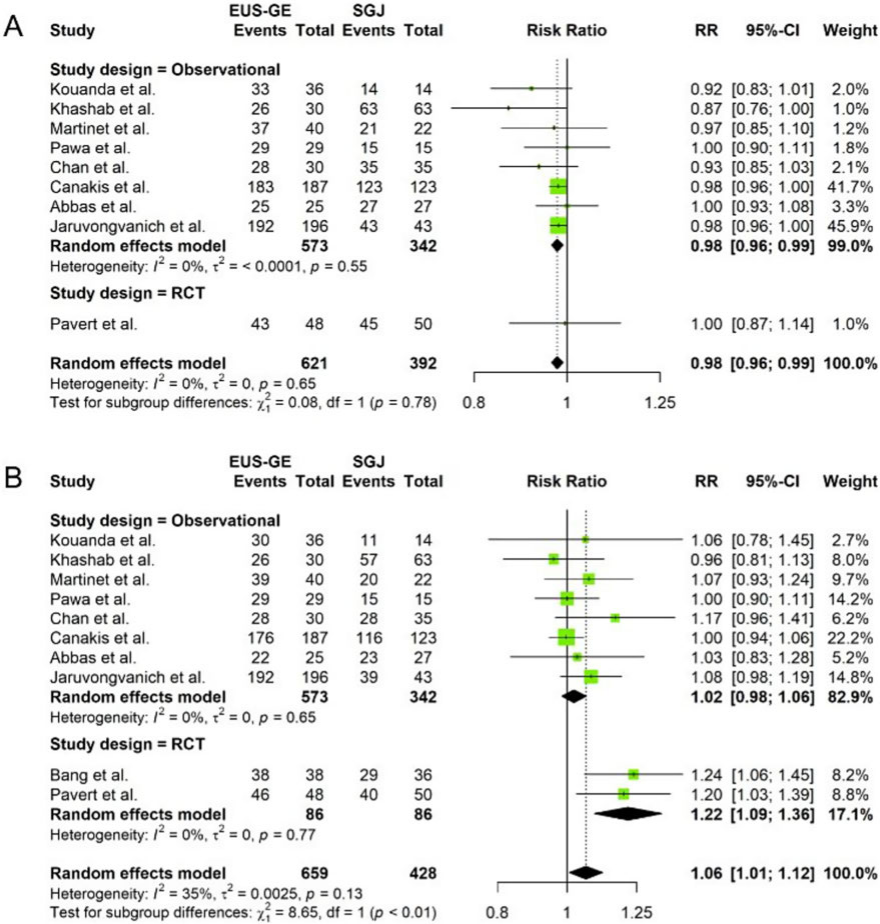
Forest plot of the meta-analysis comparing technical success ( **A** ) and clinical success ( **B** ) between EUS-GE and SGJ in patients with MGOO. EUS-GE—endoscopic ultrasound-guided gastroenterostomy; SGJ—surgical gastrojejunostomy; MGOO—malignant gastric outlet obstruction.

Fig. 2A demonstrates high and comparable technical success rates between EUS-GE and SGJ. Although the pooled effect slightly favored surgery (RR 0.98; 95% CI 0.96–0.99), the magnitude of the difference was small and of limited clinical relevance, with no observed heterogeneity ( *I* ^2^ = 0%). No significant differences were identified between observational studies and randomized trials.

EUS-GE was associated with a significantly higher clinical success rate compared with SGJ (RR 1.06; 95% CI 1.01–1.12) ( Fig. 2B ), although moderate heterogeneity was observed ( *I* ^2^ = 35%). In analyses stratified by study design, RCTs showed a larger pooled effect (RR 1.22; 95% CI 1.09–1.36) than observational studies (RR 1.02; 95% CI 0.98–1.06). A test for subgroup differences indicated that the pooled effect estimates differed significantly according to study design ( *p* < 0.01).

**Fig. 3:**
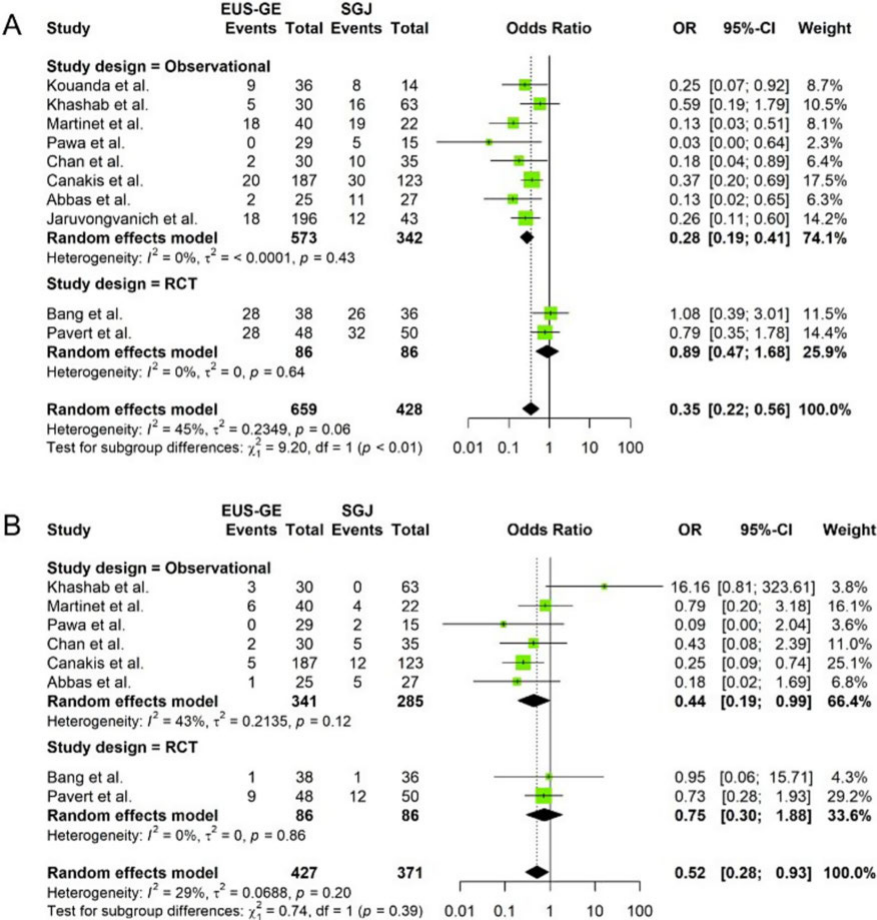
Forest plot comparing AE ( **A** ) and SAE ( **B** ) between EUS-GE and SGJ in patients with MGOO. AE—adverse events; SAE—serious adverse events; EUS-GE—endoscopic ultrasound-guided gastroenterostomy; SGJ—surgical gastrojejunostomy; MGOO—malignant gastric outlet obstruction.

Fig. 3A shows that EUS-GE was associated with a lower incidence of AE compared with SGJ (OR 0.35; 95% CI 0.22–0.56), with moderate heterogeneity ( *I* ^2^ = 45%). In analyses stratified by study design, observational studies showed a lower AE rate with EUS-GE (OR 0.28; 95% CI 0.19–0.41; *I* ^2^ = 0%), whereas RCTs showed no statistically significant difference (OR 0.89; 95% CI 0.47–1.68; *I* ^2^ = 0%). A test for subgroup differences indicated that the pooled effect estimates differed significantly according to study design ( *p* < 0.01).

EUS-GE was also associated with a lower incidence of SAE compared with SGJ (OR 0.52; 95% CI 0.28–0.93), with low heterogeneity ( *I* ^2^ = 29%). Subgroup analysis showed a more pronounced and statistically significant reduction in SAE among observational studies (OR 0.44; 95% CI 0.19–0.99), whereas randomized trials did not demonstrate a statistically significant difference (OR 0.75; 95% CI 0.30–1.88) ( Fig. 3B ).

In a sensitivity analysis excluding studies with higher risk of bias (Abbas et al. 16 and Canakis et al., 5 defined as having ≥5 moderate/serious risk domains), the association attenuated and was no longer statistically significant (OR 0.74; 95% CI 0.38–1.44).

**Fig. 4:**
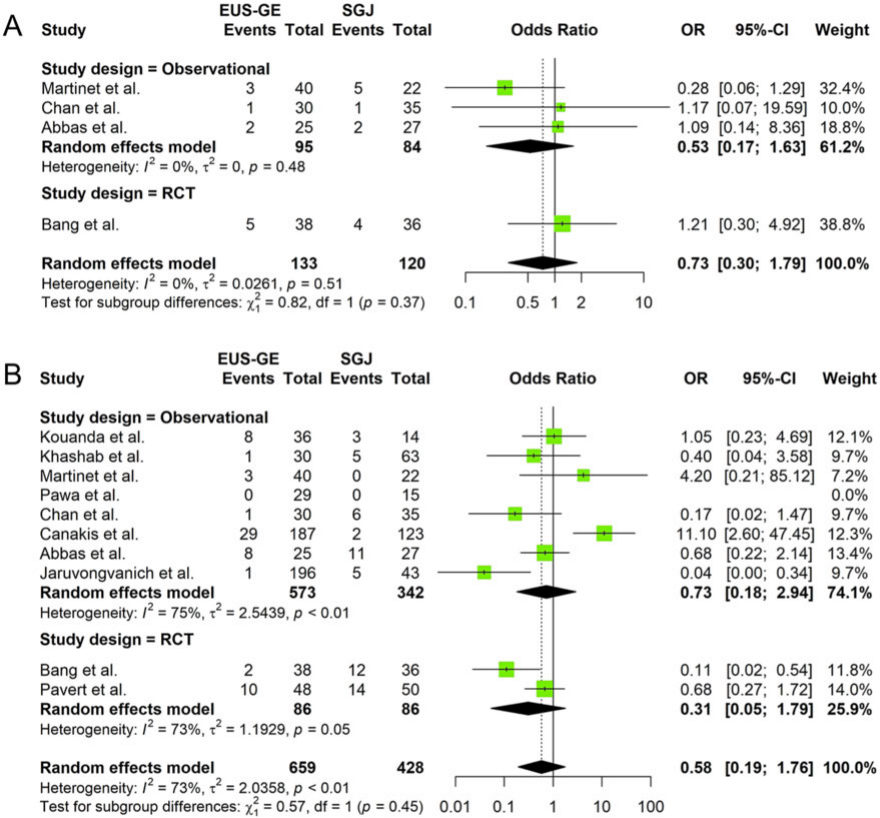
Forest plot of 30-day mortality ( **A** ) and reintervention rates ( **B** ) after EUS-GE versus SGJ in patients with MGOO. EUS-GE—endoscopic ultrasound-guided gastroenterostomy; SGJ—surgical gastrojejunostomy; MGOO—malignant gastric outlet obstruction.

The 30-day mortality was low in both groups. No statistically significant difference in mortality was observed between EUS-GE and SGJ (OR 0.73; 95% CI 0.30–1.79), with low heterogeneity across studies ( *I* ^2^ = 0%) ( Fig. 4A ).

The absolute number of reinterventions was low in both groups. In the pooled analysis, no statistically significant difference was observed between EUS-GE and SGJ regarding reintervention rates (OR 0.58; 95% CI 0.19–1.76), although substantial heterogeneity was present ( *I* ^2^ = 73%). This high heterogeneity warrants cautious interpretation, particularly considering methodological differences and sample size variability across studies ( Fig. 4B ).

In a sensitivity analysis excluding studies with a higher risk of bias (Abbas et al. 16 and Canakis et al. 5 ), EUS-GE was associated with a statistically significant reduction in reinterventions (OR 0.35; 95% CI 0.13–0.93), with reduced heterogeneity ( *I* ^2^ = 53%).

Additionally, exploratory analysis excluding only Canakis et al. 5 while retaining Abbas et al. in the pooled analysis was performed. This approach was undertaken to assess the relative contribution of each excluded study to the observed changes in the pooled estimate and heterogeneity.

Results were comparable to those observed in the sensitivity analysis excluding both Canakis et al. 5 and Abbas et al., 16 with similar pooled effect estimates and no change in the overall interpretation of the findings.

Exploratory univariable meta-regression analyses were performed to investigate potential sources of heterogeneity in the pooled treatment effect for reintervention. Study-level covariates including study design, publication year, sample size, EUS-GE technique, and risk-of-bias domains were evaluated as potential explanatory variables for the log OR. None of the tested covariates showed a statistically significant association with the estimated treatment effect.

**Fig. 5:**
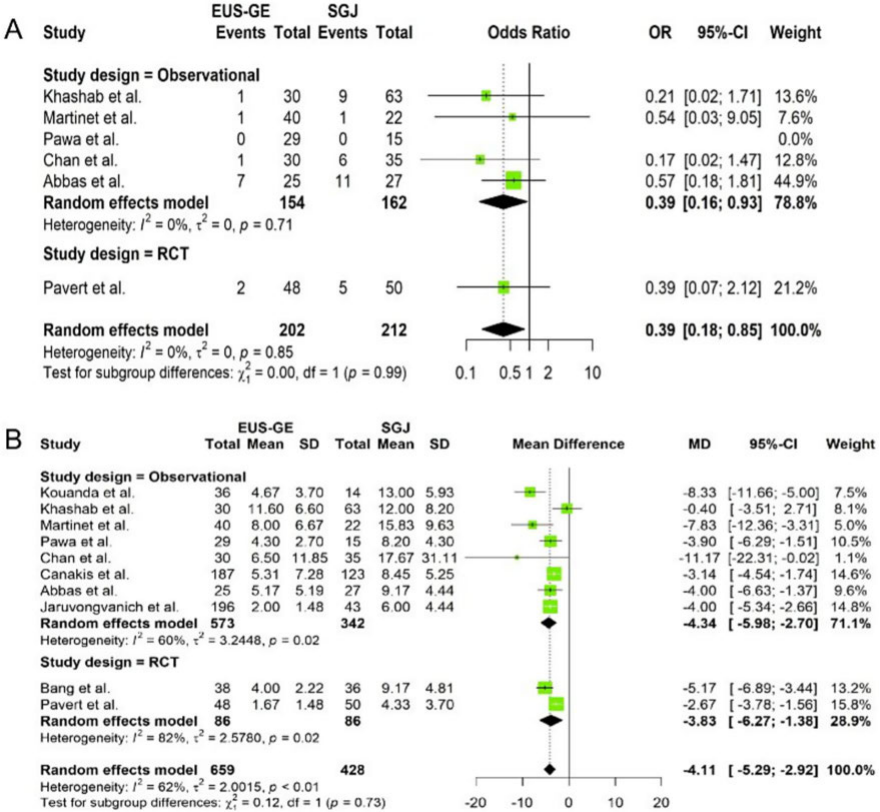
Forest plot comparing symptom recurrence ( **A** ) and LOS ( **B** ) between EUS-GE and SGJ in patients with MGOO. LOS—length of hospital stay; EUS-GE—endoscopic ultrasound-guided gastroenterostomy; SGJ—surgical gastrojejunostomy; MGOO—malignant gastric outlet obstruction.

EUS-GE was associated with a lower rate of symptom recurrence compared with SGJ (OR 0.39; 95% CI 0.18–0.85), with no significant heterogeneity ( *I* ^2^ = 0%). Subgroup analyses showed favorable effects of EUS-GE in both observational studies and randomized trials. A test for subgroup differences indicated no significant differences in pooled effect estimates according to study design ( *p* = 0.99) ( Fig. 5A ).

LOS was significantly shorter in the EUS-GE group compared with the SGJ group, with a pooled mean difference of −4.11 days (95% CI −5.29 to −2.92). Moderate to high heterogeneity was observed ( *I* ^2^ = 62%; *p* < 0.01) ( Fig. 5B ).

Visual inspection of funnel plots and Egger’s tests did not suggest relevant asymmetry for most outcomes. However, possible small-study effects or publication bias were observed for AEs and LOS, and the clinical success analysis showed borderline Egger asymmetry.

## Discussion

Patients with MGOO and peritoneal carcinomatosis may be referred to for either EUS-GE or SGJ to restore gastrointestinal continuity. Given the less invasive nature of the endoscopic approach, there is a tendency for patients with more advanced diseases, poorer clinical condition, significant comorbidities, and malnutrition to be preferentially selected for EUS-GE. 5 16 17 Despite the inclusion of more clinically complex patients in the EUS-GE group, both techniques demonstrated high technical and clinical success rates. 4 5 6 16 17 18 19 20 21 Improved oral intake capacity represents a relevant clinical outcome, as it directly reflects the patient’s functional recovery and symptom relief.

EUS-GE was associated with fewer overall and SAE compared with SGJ. 2 4 5 6 16 18 In the EUS-GE group, stent misemployment represents the most feared AE, 4 6 22 whereas stent occlusion is rarely observed, both with 15-mm and 20-mm diameter stents. 2 SAEs are uncommon and tend to decrease as operator experience increases, reflecting the learning curve associated with the EUS-GE technique. 6 20

In the SGJ group, the most common AEs include postoperative ileus/gastroparesis, infection, and anastomotic fistula. 2 Rates of infection and postoperative ileus are lower following EUS-GE compared with SGJ. These findings are likely explained by the less invasive nature of EUS-GE when compared with SGJ. 17 22

A limitation of this study is the heterogeneity in adverse AE reporting across included studies, particularly due to the use of different classification systems, such as the Clavien–Dindo classification 9 and the ASGE lexicon, 10 which are not directly interchangeable. Due to inconsistent and limited reporting, sensitivity analyses stratified by AE classification were not feasible. This variability may have influenced pooled estimates and limits the comparability of safety outcomes; therefore, results related to AE and SAE should be interpreted with caution. Although our findings suggest a potential reduction in AE and SAE with EUS-GE, these results should be considered indicative of a trend toward improved safety rather than definitive evidence. Future studies adopting standardized reporting frameworks would allow for more robust comparative and sensitivity analyses.

The 30-day mortality rate is similar between the two techniques 5 20 22 24 and type of intervention (EUS-GE or SGJ) did not affect survival when other relevant variables were factored in. 23

Several studies have demonstrated that EUS-GE is associated with shorter LOS 16 17 18 19 20 and lower symptom recurrence 16 19 when compared with SGJ, while maintaining similar and low reintervention rates. 22 23

Reinterventions in the surgical group were predominantly related to disease progression, anastomotic stricture, postoperative adhesions, and jejunogastric intussusception. 2 In the group treated endoscopically, reinterventions are mostly related to the stent. Patients with a history of previous gastrointestinal surgery have an independent risk factor for the need for further intervention. 25

A multicenter RCT including 98 patients with MGOO showed that EUS-GE was associated with a shorter time to resumption of solid oral intake and reduced LOS compared with SGJ. These findings are likely attributable to the less invasive nature of endoscopic therapy, which avoids the need for general anesthesia and surgical access to the abdominal cavity. Moreover, earlier restoration of oral intake in the endoscopic group may have contributed to faster clinical recovery and earlier hospital discharge. 26

Similarly, another multicenter randomized study involving 74 patients with MGOO demonstrated the superiority of the endoscopic approach over SGJ in terms of earlier oral intake, lower need for reinterventions, shorter hospitalization, earlier initiation or resumption of chemotherapy, improved health-related quality of life, and lower overall treatment costs. 27 Collectively, these advantages may translate into meaningful improvements in patient-centered outcomes and help reduce anxiety associated with delays in oncologic therapy.

There is no gold standard technique used in EUS-GE. A systematic review and meta-analysis of 20 studies analyzed data from 863 patients who underwent EUS-GE. Patients underwent the following techniques: DGE ( *n* = 718), BAGE ( *n* = 27), and EBOG ( *n* = 118). DGE had significantly lower rates of AEs and LOS, which can be inferred as a safer procedure. However, with the correct execution, any of the analyzed techniques may be used to palliate MGOO with similar technical and clinical outcomes. There is a learning curve for each EUS-GE technique, and the best approach should be individualized, considering personal and local expertise and availability of material and devices. 28

Palliation of MGOO in the setting of peritoneal carcinomatosis is challenging. EUS-GE has been increasingly utilized in recent years, driven by device advancements, improved endoscopic techniques, and several studies have demonstrated its favorable outcomes and safety risk profile. Standardization of techniques, improved training, and multidisciplinary collaboration are essential for the safe and successful adoption of EUS-GE in clinical practice. 2 4 In today’s healthcare system, cost savings have become an important aspect in directing treatment options. EUS-GE has the advantage of having a lower cost involved than SGJ. 21 24

Evidence comparing surgical approaches suggests that minimally invasive techniques, including laparoscopic and robotic surgery, are associated with improved perioperative outcomes such as reduced blood loss, LOS, and faster recovery compared to open surgery, while maintaining similar clinical efficacy. However, these findings are largely derived from heterogeneous surgical populations and procedures, with limited data specifically addressing gastrojejunostomy for MGOO. 29

Operator experience and the learning curve likely play a critical role in the outcomes of EUS-GE. Previous studies have demonstrated that procedural proficiency is typically achieved after approximately 25 cases, with mastery requiring up to 40 procedures, and that most AE tend to occur during the early phase of the learning curve. These findings suggest that outcomes, particularly safety, may improve with increasing operator experience and procedural standardization. 30 Therefore, more recent studies may have more favorable results.

AE reporting across studies was heterogeneous, with the use of different classification systems. The Clavien–Dindo classification, commonly used in surgical studies, grades complications according to the type of intervention required, 9 whereas the ASGE lexicon was specifically designed for endoscopic procedures and incorporates clinical severity, need for hospitalization, and procedure-related context. 10 Although both systems aim to standardize reporting, their differences in structure and clinical focus may limit comparability across studies evaluating distinct interventions. The lack of consistent use of the ASGE lexicon represents a limitation, as it hindered a procedure-specific and standardized assessment of AE and may have contributed to heterogeneity and potential misclassification bias in the pooled analysis.

This study is limited by the predominance of retrospective data, the inclusion of patients with heterogeneous clinical profiles, limited follow-up duration, and changes in practice patterns over time, as well as the fact that most studies were conducted in specialized, high-complexity centers. In addition, the number of observational studies and their corresponding sample size substantially exceeded those of RCTs, which may reduce the robustness of the conclusions. Clinical heterogeneity is also reflected in variations among EUS-GE techniques, as the procedure lacks standardization. Moreover, operator experience was not consistently reported or adjusted for, which may have influenced procedural outcomes. Furthermore, larger 20-mm stents have only recently become available.

Given the number of secondary outcomes, subgroup analyses, sensitivity analyses, and exploratory meta-regressions performed, no formal adjustment for multiple comparisons was applied. Therefore, secondary and exploratory findings should be interpreted cautiously, particularly those with borderline statistical significance.

Despite these limitations, our study has several strengths, including the large, pooled sample size, inclusion of recent studies reflecting current practices, and comprehensive analysis of clinically relevant outcomes.

The EUS-GE technique is still in early phases of development and will continue to evolve with the advent of purpose-built accessories to improve the efficiency and safety of this technique. Further studies are needed to address the optimal technique to maximize a safe and successful LAMS placement. A future analysis could explore the EUS-GE technique, which may yield better results at a lower cost.

## Conclusion

In this systematic review and meta-analysis, both EUS-GE and SGJ demonstrated high technical success and low mortality and reintervention rates for the palliative management of MGOO. However, EUS-GE was associated with modestly higher clinical success, shorter LOS, lower symptom recurrence, and a significantly reduced incidence of AE and SAE.

These findings suggest that EUS-GE represents a safe and effective minimally invasive alternative to SGJ, particularly in patients with advanced malignancy who may benefit from faster recovery and reduced procedural morbidity.

Future high-quality randomized trials and standardized outcome reporting are needed to further define patient selection criteria and to establish the optimal position of EUS-GE in clinical guidelines.

**Abbreviations**
MGOO: malignant gastric outlet obstruction
SGJ: surgical gastrojejunostomy
EUS-GE: endoscopic ultrasound-guided gastroenterostomy
LAMS: lumen-apposing metal stents
RCTs: randomized controlled trials
AE: adverse events
GOOSS: gastric outlet obstruction scoring system
LOS: length of hospital stay
GRADE: grading of recommendations assessment, development and evaluation
RRs: risk ratios
ORs: odds ratios
SAE: serious AEs
MDs: mean differences
CIs: confidence intervals (CIs)
IQR: interquartile range.

## References

[1] Papanikolaou I S Siersema P D Gastric outlet obstruction: current status and future directions Gut Liver 2022 16 05 667 675 10.5009/gnl210327 35314520 PMC9474481

[2] Jaruvongvanich V Mahmoud T Abu Dayyeh B K Endoscopic ultrasound-guided gastroenterostomy for the management of gastric outlet obstruction: a large comparative study with long-term follow-up Endosc Int Open 2023 11 01 E60 E66 10.1055/a-1976-2279 36644538 PMC9839427

[3] Koop A H Palmer W C Stancampiano F F Gastric outlet obstruction: a red flag, potentially manageable Cleve Clin J Med 2019 86 05 345 353 10.3949/ccjm.86a.18035 31066665

[4] Teoh A YB Chan S M Yip H C Is endoscopic ultrasound-guided gastroenterostomy better than surgical gastrojejunostomy or duodenal stenting? Dig Endosc 2025 37 01 77 84 10.1111/den.14929 39370536 PMC11718137

[5] Canakis A Bomman S Lee D U Benefits of EUS-guided gastroenterostomy over surgical gastrojejunostomy in the palliation of malignant gastric outlet obstruction: a large multicenter experience Gastrointest Endosc 2023 98 03 348 3.59E32 10.1016/j.gie.2023.03.022 37004816

[6] Miller C Benchaya J A Martel M EUS-guided gastroenterostomy vs surgical gastrojejunostomy and enteral stenting for malignant gastric outlet obstruction: a meta-analysis Endosc Int Open 2023 11 07 E660 E672 10.1055/a-2098-2570 37593104 PMC10431974

[7] Page M J McKenzie J E Bossuyt P M The PRISMA 2020 statement: an updated guideline for reporting systematic reviews BMJ 2021 372 n71 10.1136/bmj.n71 33782057 PMC8005924

[8] Adler D G Baron T H Endoscopic palliation of malignant gastric outlet obstruction using self-expanding metal stents: experience in 36 patients Am J Gastroenterol 2002 97 01 72 78 10.1111/j.1572-0241.2002.05423.x 11808972

[9] Dindo D Demartines N Clavien P A Classification of surgical complications: a new proposal with evaluation in a cohort of 6336 patients and results of a survey Ann Surg 2004 240 02 205 213 10.1097/01.sla.0000133083.54934.ae 15273542 PMC1360123

[10] Cotton P B Eisen G M Aabakken L A lexicon for endoscopic adverse events: report of an ASGE workshop Gastrointest Endosc 2010 71 03 446 454 10.1016/j.gie.2009.10.027 20189503

[11] Yang C Y Huang W H Cheng H H Endoscopic ultrasound-guided gastroenterostomy, with focus on technique and practical tips Clin Endosc 2025 58 02 201 217 10.5946/ce.2024.206 40200658 PMC11982821

[12] Sterne J AC Savović J Page M J RoB 2: a revised tool for assessing risk of bias in randomised trials BMJ 2019 366 l4898 10.1136/bmj.l4898 31462531

[13] Sterne J A Hernán M A Reeves B C ROBINS-I: a tool for assessing risk of bias in non-randomised studies of interventions BMJ 2016 355 i4919 10.1136/bmj.i4919 27733354 PMC5062054

[14] Guyatt G H Oxman A D Vist G E GRADE: an emerging consensus on rating quality of evidence and strength of recommendations BMJ 2008 336 7650 924 926 10.1136/bmj.39489.470347.AD 18436948 PMC2335261

[15] Wan X Wang W Liu J Estimating the sample mean and standard deviation from the sample size, median, range and/or interquartile range BMC Med Res Methodol 2014 14 135 10.1186/1471-2288-14-135 25524443 PMC4383202

[16] Abbas A Dolan R D Bazarbashi A N Endoscopic ultrasound-guided gastroenterostomy versus surgical gastrojejunostomy for the palliation of gastric outlet obstruction in patients with peritoneal carcinomatosis Endoscopy 2022 54 07 671 679 10.1055/a-1708-0037 35120397

[17] Pawa R Koutlas N J Russell G Endoscopic ultrasound-guided gastrojejunostomy versus robotic gastrojejunostomy for unresectable malignant gastric outlet obstruction DEN Open 2023 4 01 e248 10.1002/deo2.248 37228709 PMC10204173

[18] Bronswijk M Vanella G van Malenstein H Laparoscopic versus EUS-guided gastroenterostomy for gastric outlet obstruction: an international multicenter propensity score-matched comparison Gastrointest Endosc 2021 94 03 526 53600 10.1016/j.gie.2021.04.006 33852900

[19] Chan S M Dhir V Chan Y YY Endoscopic ultrasound-guided balloon-occluded gastrojejunostomy bypass, duodenal stent or laparoscopic gastrojejunostomy for unresectable malignant gastric outlet obstruction Dig Endosc 2023 35 04 512 519 10.1111/den.14472 36374127

[20] Martinet E Gonzalez J M Thobois M Surgical versus endoscopic gastroenterostomy for gastric outlet obstruction: a retrospective multicentric comparative study Langenbeck’s Arch Surg 2024 409 01 192 10.1007/s00423-024-03365-1 38900214

[21] Perez-Miranda M Tyberg A Poletto D EUS-guided gastrojejunostomy versus laparoscopic gastrojejunostomy: an international collaborative study J Clin Gastroenterol 2017 51 10 896 899 10.1097/MCG.0000000000000887 28697151

[22] Kouanda A Binmoeller K Hamerski C Endoscopic ultrasound-guided gastroenterostomy versus open surgical gastrojejunostomy: clinical outcomes and cost effectiveness analysis Surg Endosc 2021 35 12 7058 7067 10.1007/s00464-020-08221-z 33479837

[23] Khashab M A Bukhari M Baron T H International multicenter comparative trial of endoscopic ultrasonography-guided gastroenterostomy versus surgical gastrojejunostomy for malignant gastric outlet obstruction Endosc Int Open 2017 5 04 E275 E281 10.1055/s-0043-101695 28382326 PMC5378550

[24] Pinnam B SM Ojemolon P E Mohammed A Comparison of endoscopic and surgical gastrojejunostomy in patients with malignant gastric outlet obstruction: a national cohort analysis Gastrointest Endosc 2025 102 02 205 2130 10.1016/j.gie.2025.01.025 39870244

[25] Pasam R T Mathews T Schuster K F EUS-guided gastroenterostomy for malignant gastric outlet obstruction: impact of clinical and demographic factors on outcomes Gastrointest Endosc 2025 101 03 580 5880 10.1016/j.gie.2024.10.053 39491733 PMC12091699

[26] van de Pavert Y L Endoscopic versus surgical gastroenterostomy for palliation of malignant gastric outlet obstruction (ENDURO): a randomised controlled trial Lancet Gastroenterol Hepatol 2025 10 12 1065 1074 41061719 10.1016/S2468-1253(25)00209-2

[27] Bang J Y Puri R Lakhtakia S Endoscopic or surgical gastroenterostomy for malignant gastric outlet obstruction: a randomised trial Gut 2025 75 01 24 32 10.1136/gutjnl-2025-336339 40998416 PMC12703243

[28] Ribas P HBV De Moura D TH Proença I M Endoscopic ultrasound-guided gastroenterostomy for the palliation of gastric outlet obstruction: a systematic review and meta-analysis of the different techniques Cureus 2022 14 11 e31526 10.7759/cureus.31526 36540454 PMC9754671

[29] Marano A Choi Y Y Hyung W J Robotic versus laparoscopic versus open Gastrectomy: a meta-analysis J Gastric Cancer 2013 13 03 136 148 10.5230/jgc.2013.13.3.136 24156033 PMC3804672

[30] Jovani M Ichkhanian Y Parsa N Assessment of the learning curve for EUS-guided gastroenterostomy for a single operator Gastrointest Endosc 2021 93 05 1088 1093 10.1016/j.gie.2020.09.041 32991868

