# Correction: Endoscopic or Surgical Gastroenterostomy for Malignant Gastric Outlet Obstruction: A Systematic Review and Meta-analysis

**DOI:** 10.1055/a-2917-7228

**Published:** 2026-08-07

**Authors:** Leonardo Marinho, Alessandrino T. de Oliveira, Márcio A. Barreira, Erica U. Holanda, Ana C. de Oliveira Monteiro

**Affiliations:** 1 Postgraduate Studies in Medical Sciences 28128 Universidade de FortalezaFortalezaCEBrazil; 2 Department of Surgery 633701 Dr José Frota InstituteFortalezaCEBrazil; 3 Department of Digestive Endoscopy 365090 General Hospital of FortalezaFortalezaCearáBrazil; 4 Department of Surgery 293278 Universidade Federal do Ceara Hospital Universitario Walter CantidioFortalezaCearáBrazil; 5 Department of Internal Medicine 672398 Hospital Geral Dr Waldemar AlcântaraFortalezaCEBrazil



In the above-mentioned article the spelling of “Gastroenterostomy” in the title has been corrected.

This was corrected in the online version on July 23, 2026.

